# Prevalence vs impact: a mixed methods study of survivorship issues in colorectal cancer

**DOI:** 10.1007/s11136-021-02975-2

**Published:** 2021-08-21

**Authors:** Amanda Drury, Sheila Payne, Anne-Marie Brady

**Affiliations:** 1 School of Nursing, Midwifery and Health Systems, University College Dublin, Belfield, Dublin 4, Ireland; 2 Division of Health Research, International Observatory on End of Life Care, Lancaster University, Lancaster, LA1 4AT UK; 3 School of Nursing & Midwifery, Faculty of Health Sciences, Trinity College Dublin, 24 DʹOlier Street, Dublin 2, D02 T283 Ireland

**Keywords:** Colorectal cancer, Cancer survivorship, Patient reported outcomes, Mixed methods, Integration

## Abstract

**Purpose:**

This study aims to explore the prevalence of CRC survivorship issues and their impact on survivors’ quality of life (QoL).

**Methods:**

This study utilised a mixed methods sequential explanatory design. Adult CRC survivors between 6- and 60-months post-diagnosis (*n* = 304) were purposively recruited from three hospitals and twenty-one cancer support centres in Ireland. QoL was evaluated using the EuroQol and FACT-C questionnaires and results compared to population norms. 22 survey participants took part in semi-structured interviews exploring the impact of survivorship issues on their daily lives.

**Results:**

While CRC survivors reported QoL outcomes comparable to or better than normative populations, 54% were dissatisfied with their QoL. The most common survivorship issues reported included negative body image (74%), fatigue (68%), sexual dysfunction (66%) and sleep disturbance (59%). Thematic analysis of the qualitative data illustrated survivors’ attempts to *live with* the impact of cancer and its treatment (loss, fear, impact) and *striving* to contextualise, reframe and understand the consequences of cancer and its treatment (control, vigilance, benefit). Within these themes, the cross-domain impact of less prevalent symptoms including bowel dysfunction (28–57%) and peripheral neuropathy (47%) were widely discussed.

**Conclusions:**

Although cancer survivors report positive QoL outcomes, many experience distressing physical, psychological and social effects. The findings suggest less common and difficult to manage symptoms are the greatest source of distress and unmet need. Support and information must be tailored to address survivors’ individual needs and preferences for support, informed by holistic person-centred assessment.

**Supplementary Information:**

The online version contains supplementary material available at 10.1007/s11136-021-02975-2.

## Plain English summary

People living after cancer treatment may experience symptoms and ill-health as a result of their cancer and its treatment, for a long time after treatment. People living after cancer experience a range of emotions, including anxiety and fears about their well-being, their treatment and their family. In this study we explore the physical and psychological problems experienced by people living after colorectal cancer, and how these problems affect their quality of life (QoL). This study found that while people living after colorectal cancer reported QoL levels comparable to members of the general population, more than half were dissatisfied with their QoL. Findings suggest less prevalent cancer-related problems were more likely to negatively affect QoL outcomes. Findings from this study highlight that support for people living after cancer must be guided by the needs and preferences of the individual with cancer, rather than the most common problems experienced by this group.

## Introduction

Quality of Life (QoL) is complex; it is highly individual, influenced by values, expectations and cultures, manifesting in an individuals’ perception of their physical, psychological, social and functional well-being [1, 2]. Colorectal cancer (CRC) survivors’ QoL may improve over time, and for some, may reach levels that are comparable to normative populations and pre-diagnosis [3–5]. Despite this, a substantial proportion of CRC survivors may experience distressing physical, psychological and social effects, persisting for years beyond diagnosis [6–8]. Adding to the complexity of understanding the needs of cancer survivors is the wide variation in the reported prevalence of various physical, psychological and social issues (Supplementary Appendix 1) [9].

The transition to life after cancer treatment presents various challenges for cancer survivors, with variable rates of adjustment and recovery [10, 11]. Previous studies highlight the adverse impact of an ostomy and physical effects of cancer on QoL outcomes, including fatigue, pain, sexual dysfunction and bowel dysfunction [8, 12–16]. Furthermore, cancer survivors may experience significant psychological distress related to fear of recurrence [10–12, 14, 15, 17]. Qualitative studies describe the impact of response shift, post-traumatic growth and enhanced coping capabilities within the process of cancer recovery and survivorship [8, 10, 11, 18]. Nevertheless, many cancer survivors express persistent unmet supportive care needs to address physical and psychosocial consequences of cancer [17, 19–21]. Cancer survivorship represents a new normal, requiring significant adjustment and time to identify, implement and master self-management techniques [11, 16, 17, 20, 22].

The diversity of methodologies used in QoL research for CRC survivors is dominated by quantitative approaches, with a small, but growing number of qualitative and mixed methods studies. Quantitative methods serve an important role in highlighting the prevalence of survivorship issues and factors which may influence QoL in cancer survivorship. However, studies of this nature may make recommendations for cancer care services based on prevalence of survivorship issues, overlooking less common effects, and individual needs and preferences of cancer survivors. Mixed methods research is increasingly recognized as an appropriate method of enquiry to understand the complexity of chronic illness, yet integration is often overlooked at the interpretation and reporting stage [23]. While efforts to achieve integration at design and methods level are evident in the CRC survivorship literature, the results of mixed methods studies may be presented separately with limited consideration of their relationship to results drawn from the alternative method [24–26]. This is consistent with practices in other fields [23, 27]. This study therefore aims to explore the prevalence and impact of CRC survivorship issues on survivors’ QoL using a sequential mixed methods approach. A secondary objective of this study was to compare dimensions of QoL among CRC survivors with those of normative populations. The quantitative phase of the study presents the prevalence of physical, psychological and social survivorship issues, and their association with QoL outcomes. The qualitative phase of this study was designed to build upon the quantitative phase, to provide further context and understanding of CRC survivors’ experiences of survivorship issues, and how they influence QoL outcomes. Integration of methods and analysis within this study will provide a more comprehensive understanding of the processes by which cancer survivorship issues may influence QoL outcomes among CRC survivors.

## Materials and methods

### Design

A pragmatic mixed-methods sequential explanatory design guided *The Cost of Survival Study* [28]. The methods utilised in the quantitative and qualitative phases of this study are reported in detail elsewhere [24, 25]. CRC survivors aged 18 years or older, of any disease stage, between 6- and 60-months post-diagnosis, and undergoing surveillance and follow up care were recruited to the quantitative questionnaire study (Phase 1) via surgical and medical oncology clinics in three public and private hospitals and 21 voluntary cancer support centres.

A subsample of Phase 1 participants were invited to take part in qualitative semi-structured interviews (Phase 2). Purposive recruitment of participants to Phase 2 was guided by the results of the quantitative phase of the study [24]; a maximum variation sampling strategy was designed to achieve representation of variations in QoL outcomes (*high/low FACT-C score*, based on sample median score of 116), and variables which were associated with CRC survivors’ QoL outcomes in Phase 1. These included demographic (*age, gender living arrangements, employment status*), health (*diagnosis, disease status, time since diagnosis, chemotherapy, presence of an ostomy, comorbidities*) and healthcare-related characteristics (*private health insurance, type of hospital attended, level of satisfaction with continuity of care, treatment summary, access to a named nurse for cancer-related worries, access to a named doctor for cancer-related worries, feeling supported by community services staff, accessed voluntary cancer support services, social difficulties, unmet information needs*). Phase 2 recruitment continued until data saturation was achieved.

The study received ethical approval from the Research Ethics Committees of the School of Nursing and Midwifery, Trinity College Dublin (Reference: The Cost of Survival) and participating hospitals (Reference: 2014/05 Chairman’s Action [6]; Reference: 06/2014 The Cost of Survival).

### Data collection

In the first, quantitative phase of this study, participants completed subjective measures of QoL and social difficulties. There are numerous measurement scales which assess physical, psychological and social functioning across a variety of populations, with significant overlap in the content of these measures [29]. To ensure that a full picture of CRC survivors’ QoL and symptom experience were captured with minimal overlap, a content analysis of commonly used QoL instruments was undertaken, including generic (*Short Form-36; EuroQOL 5D-5L*), cancer-specific [*Functional Assessment Cancer Therapy-General (FACT-G), European Organisation for Research and Treatment of Cancer quality of life questionnaire (EORTC-QLQ-C30*)], and CRC specific [*FACT-Colorectal (FACT-C), EORTC QLQ-CR29 & QLQ-CR38*] measures. Following content analysis and consultation with clinical and academic experts in CRC and QoL, and evaluation of quantitative and qualitative empirical literature describing CRC survivors’ QoL and symptom experiences, the EuroQOL 5D-5L and FACT-C were selected for inclusion in the questionnaire. The EuroQol ED-5L [30] evaluates health in five dimensions (mobility, self-care, usual activities, pain/discomfort and anxiety/depression) at five levels of severity (no problem; slight problem, moderate problem, severe problem and unable to complete). The EuroQol Visual Analogue Scale (VAS) evaluates self-rated health on ranging from 0, “*the worst health you can imagine*” to 100, “*the best health you can imagine*”. The Functional Assessment of Chronic Illness Therapy–Colorectal (FACT-C) [31] measures QoL in five domains [Physical well-being (PWB), Social well-being (SWB), Emotional well-being (EWB), Functional well-being (FWB) and Colorectal Cancer Concerns (CCS)]. Items are assessed using a Likert-scale format, with item scores ranging from 0, *not at all* to 4, *very much*. The FACT-General score may be calculated from the sum of PWB, SWB, EWB and FWB items (range 0–108); the FACT-C score is the sum of the FACT-G and CCS scores (range 0–136).

Additional items representing survivorship issues reported in the empirical literature, were identified. To ensure these issues were captured, the Social Difficulties Inventory (SDI) [32] was included, providing insight to social issues experienced by cancer patients in three domains; (1) Everyday Living (EDL), (2) Money Matters (MM) and (3) Self and Others (SO). The SDI consists of 21 Likert-scale items with responses ranging from 0, *no difficulty* to 3, *very much*. Additional physical and functional survivorship issues that were identified in the CRC literature, but were not components of the selected instruments, were generated based on the National Cancer Institute Common Terminology Criteria for Adverse Events [33]. Generated symptom items included constipation, peripheral neuropathy, and cognitive changes, and were treated as stand-alone items.

While QoL outcomes are relevant and useful in and of themselves, their value in the context of chronic illness is enhanced if they are interpreted in the context of population norms [3, 34]. The use of age- and gender-matched normative data are preferable, however, the comparison of the sample distribution with that of a normative reference group nevertheless provides a valuable benchmark for comparison [34]. To provide a more comprehensive understanding of CRC survivors’ QoL outcomes, EuroQOL VAS and FACT-C scores were compared with previously published normative population data. Normative data for the FACT-C is not available for Ireland; therefore, FACT-C scores for the current sample are compared to normative population scores for the USA (*n* = 1075; 50.6% female, 15.5% ≥ 65 years) [34] and Austria (*n* = 926; 48.3% female, 14.7% ≥ 60 years) [3]. EuroQOL VAS scores and health dimensions for the current sample are compared to normative population scores in Ireland (*n* = 1131; 62% female, 22% ≥ 65 years) [35] and the UK (*n* = 3395; 57% female, 22% ≥ 65 years) [36, 37].

Semi-structured telephone interviews were conducted with CRC survivors to explore their experiences of QoL in cancer survivorship. The interview schedule was designed to probe quantitative findings further, providing complementary information which could contextualise the quantitative results, providing insight into the experience of survivorship issues, and how they affect survivors’ daily life. Participants were asked to describe their experiences of cancer survivorship and their QoL via the broad question, *‘Could you please tell me about your experiences of living with/after colon/rectal cancer?’*. Probing questions were designed based on the domains of QoL and the theoretical framework which guided this study [38], exploring the physical, psychological and social survivorship issues identified in Phase 1 in greater depth:

- Tell me about the main symptoms/side-effects you have experienced since completing your initial cancer treatment?
- How do these symptoms affect your daily life?
- With regard to your cancer, could you tell me about the worries you have for you or your family?
- How have your family and friends helped you since completing your initial cancer treatment?
- Has having cancer had any impact on your relationships with your family and friends?
- Could you tell me about any positive changes or experiences in your life since completing your cancer treatment?
- Could you tell me about any negative changes or experiences in your life since completing your cancer treatment?

Interviews lasted between 35 and 110 minutes and were audio-recorded and transcribed verbatim.

### Rigor

Methodological rigour in this study was ensured with the use of strategies to address the standards of quality appraisal for mixed methods research; veracity, consistency, applicability and neutrality (Curry 2015). All standardised surveys employed in the quantitative study (EuroQOL, FACT-C, SDI) had established validity and reliability with English-speaking samples of people living with cancer [30] or CRC [31, 39], and demonstrated acceptable levels of internal consistency for the current sample (*α* ≥ 0.70) [24].

Reflexive journaling, maintaining field notes and member checking of transcripts enhanced the veracity and consistency of data collection and analysis in the qualitative phase. Applicability of the findings of this study was enhanced through the engagement of participants from diverse backgrounds and research sites in a nested sample, and a maximum variation sampling strategy in Phase 2. Neutrality in this study was fostered through the transparent reporting of methods and engagement in reflexive journaling.

### Data analysis and integration

In the current study, a mixed methods approach provides an opportunity to comprehensively evaluate the scope and meaning of survivorship issues and QoL for CRC survivors. Quantitative and qualitative data were managed and analysed sequentially according to the principles of statistical (Phase 1) and thematic analysis (Phase 2).

Quantitative data were analysed descriptively using frequencies, crosstabulations and measures of central tendency (mean, standard deviation). Chi-square statistics and odds ratios were used to investigate differences between the proportion of CRC survivors reporting lower QoL between groups who reported and did not report problems with individual survivorship issues on the EuroQOL 5D-5L, FACT-C and SDI. For the purpose of this analysis, the FACT-C score was dichotomised using an a priori cut-off of the sample median (116.0). A significance level of *p* ≤ 0.05 was used. Differences in mean scores for dimensions of QoL among CRC survivors and normative populations were tested using the summary independent sample t-tests at a two-sided significance of *p* ≤ 0.05. Differences in the proportion of CRC survivors and normative populations reporting any problems with EuroQOL 5D-5L items was tested using a two-sample z-test.

Qualitative data were analysed thematically, guided by the Braun & Clarke [40] thematic analysis framework. Interview transcripts were coded inductively, transcripts were read and re-read to become familiar with the data. Initial codes were generated from the data and subsequently organised and reorganised, searching for themes and sub-themes. Themes were reviewed through a deductive re-analysis process; themes which lacked sufficient data were discarded. The final themes were named, defined and written up. Annotation, memo and link functions in NVivo were used in conjunction with reflective journaling to ensure critical reflection on the process of data collection and analysis.

Integration in this study was achieved via a contiguous approach, *connecting* quantitative and qualitative phases through a nested, integrated sampling strategy, *building* through the use of quantitative findings to underpin the sampling and data collection techniques utilised in the qualitative phase and finally, *embedding*, through the integration of quantitative and qualitative data analysis [41]. While quantitative and qualitative data were initially conducted sequentially, an iterative approach to analysis was undertaken, moving back and forth between datasets, nurturing cross-fertilisation of inferences, and supporting interrogation of emerging convergence and divergences between quantitative and qualitative data [42]. The quantitative and qualitative findings of this study are presented sequentially, highlighting the contributions of the quantitative and qualitative data; signposts at key junctures in the presentation of the results illuminate the additional context, depth, explanation and perspectives which emerged through the iterative, narrative approach to integration. Meta-inferences generated through the interpretation of integrated quantitative and qualitative findings are presented in the discussion, considered in the context of empirical literature to interpret, explain and extend understanding of CRC survivors’ experiences of survivorship issues and QoL, identifying confirmatory, explanatory or discordant results [41, 43, 44].

## Results

### Sample characteristics

The demographic characteristics of participants are presented in Table 1. Phase 1 (*N* = 304) participants ages ranged between 25 and 96 years; Phase 2 participants (*N* = 22) were between 47 and 78 years of age. Participants were diagnosed between 1 and 5 years previously; the majority were in remission at the time of the study (Phase 1: 82.9%; Phase 2: 90.9%) and reported a diagnosis of colon cancer (Phase 1: 64.1%; Phase 2: 59.1%). Participants were predominantly male (Phase 1: 55.8%; Phase 2: 54.5%), and living with a family member (Phase 1: 81.3%; Interviews: 81.8%) in urban areas (Phase 1: 76.5%; Phase 2: 54.5%).

**Table 1:** Demographic characteristics of sample

Characteristic	Response	Questionnaire (*N* = 304)		Interview (*N* = 22)	
		*n*	%	*n*	%
Demographic context variables					
Age	< 65 years	93	32.9	10	45.5
	≥ 65 years	190	67.1	12	54.5
Gender	Female	126	44.2	10	45.5
	Male	159	55.8	12	54.5
Living arrangements	Lives with others	231	81.3	18	81.8
	Lives alone	53	18.7	4	18.2
Area of residence	Urban	215	76.5	12	54.5
	Rural	66	23.5	10	45.5
Change in employment status since diagnosis	Remained/became employed	71	25.0	9	40.9
	Remained unemployed	167	58.8	9	40.9
	Became unemployed	46	16.2	4	18.2
Private health insurance	Yes	138	48.3	13	59.1
	No	148	51.7	9	40.9
Ethnicity	Irish	274	95.5	22	100.0
	Other	13	4.5	0	0.0
Diagnosis	Colon	191	64.1	13	59.1
	Rectum	69	23.2	7	31.8
	Other	38	12.8	2	9.1
Time since diagnosis	< 2 years	110	39.6	7	31.8
	≥ 2 years	168	60.4	15	68.2
Radiotherapy	No radiotherapy	228	76.0	12	54.5
	Any radiotherapy	72	24.0	10	45.5
Chemotherapy	No chemotherapy	125	41.7	3	13.6
	Any chemotherapy	175	58.3	19	86.4
Surgery	No surgery	26	8.7	2	9.1
	Any surgery	273	91.3	20	90.9
Stoma	Never had a stoma	153	53.5	10	45.5
	Stoma reversed	78	27.3	7	31.8
	Stoma present	55	19.2	5	22.7
Disease status	In remission	228	82.9	20	90.9
	Any active disease	47	17.1	2	9.1
Comorbidities	None	62	22.1	4	18.2
	One or more	218	77.9	18	81.8

### QoL in CRC survivorship

On average, CRC survivors were more likely to report issues with mobility, usual activities and anxiety or depression compared to the general population in Ireland and the UK (all *p* ≤ 0.001) (Supplementary Appendix 2). Compared to Austrian FACT-G population norms, participants of this study reported higher FACT-G, SWB and EWB scores (all *p* ≤ 0.05), and comparable PWB (*p* = 0.300) and FWB (*p* = 0.610) scores on average (Supplementary Appendix 3). Compared to US population norms, participants of this study reported higher FACT-G, PWB, SWB and FWB scores (all *p* ≤ 0.05), and similar EWB scores (*p* = 0.552) (Supplementary Appendix 3). Despite these positive mean scores, more than half of survivors were dissatisfied with their QoL on the FACT-C item “*I Am Content with the Quality of My Life Right Now*” (54%) (Table 2).

**Table 2:** Prevalence of survivorship issues reported by colorectal cancer survivors and their association with poorer overall QoL outcomes

Source	Side-effect	Symptom prevalence		No problem reported with symptom		Problem reported with symptom		OR	95% confidence interval		***p***
		*n*	%	*n*	% with lower FACT-C score	*n*	% with lower FACT-C score		Lower	Upper	
EuroQOL	Mobility	85	28.8	90	42.9	66	77.6	4.632	2.596	8.263	≤ 0.001
	Self-care	18	6.1	140	50.4	17	94.4	16.757	2.200	127.647	≤ 0.001
	Engagement with usual activities	108	36.7	68	36.6	88	81.5	7.635	4.318	13.500	≤ 0.001
	Pain or discomfort	98	33.7	77	39.9	77	78.6	5.524	3.149	9.690	≤ 0.001
	Anxiety or depression	88	29.9	84	40.8	71	80.7	6.066	3.337	11.028	≤ 0.001
PWB	Fatigue	197	68.4	17	18.7	132	67.0	8.840	4.827	16.189	≤ 0.001
	Nausea	33	13.3	91	42.3	31	93.9	21.121	4.928	90.517	≤ 0.001
	Meeting family needs	60	22.9	78	38.6	54	90.0	14.308	5.877	34.831	≤ 0.001
	Pain	79	30.3	62	34.1	70	88.6	15.054	7.049	32.148	≤ 0.001
	Problem with side-effects	91	34.6	68	39.5	65	71.4	3.824	2.210	6.614	≤ 0.001
	Feeling ill	32	12.3	100	43.7	31	96.9	39.990	5.367	297.973	≤ 0.001
	Bed bound	58	22.0	82	39.8	53	91.4	16.029	6.147	41.798	≤ 0.001
SWB	Close to friends	120	43.0	49	30.8	91	75.8	7.044	4.119	12.048	≤ 0.001
	Emotional support from family	94	34.3	64	35.6	75	79.8	7.155	3.971	12.890	≤ 0.001
	Support from friends	129	47.8	38	27.0	98	76.0	8.569	4.948	14.838	≤ 0.001
	My family have accepted my illness	56	20.0	90	40.2	53	94.6	26.304	7.975	86.761	≤ 0.001
	Satisfaction with communication about illness	67	24.3	80	38.3	60	89.6	13.821	6.021	31.729	≤ 0.001
	Feeling close to partner	51	23.0	67	39.2	41	80.4	6.364	2.987	13.559	≤ 0.001
	Satisfied with sexual function	102	65.8	18	34.0	63	61.8	3.141	1.568	6.292	0.001
EWB	Feeling sad	116	42.0	46	28.8	90	77.6	8.579	4.926	14.938	≤ 0.001
	Satisified with cancer-related coping	126	43.8	55	34.0	92	73.0	5.264	3.160	8.770	≤ 0.001
	Losing hope	21	7.6	116	45.7	21	100.0	1.181	1.100	1.268	≤ 0.001
	Feeling nervous	110	40.1	58	35.4	78	70.9	4.455	2.645	7.503	≤ 0.001
	Worried about death	121	43.7	60	38.5	79	65.3	3.010	1.836	4.933	≤ 0.001
	Worry about worsening condition	143	51.3	51	37.5	91	63.6	2.917	1.793	4.744	≤ 0.001
FWB	Able to work	157	56.5	23	19.0	117	74.5	12.463	6.986	22.234	≤ 0.001
	Work is fulfilling	152	57.1	22	19.3	109	71.7	10.600	5.912	19.007	≤ 0.001
	Able to enjoy life	133	47.2	31	20.8	111	83.5	19.205	10.490	35.160	≤ 0.001
	Accepted illness	88	31.3	70	36.3	70	79.5	6.833	3.768	12.393	≤ 0.001
	Sleeping well	167	59.0	31	26.7	112	67.1	5.584	3.310	9.417	≤ 0.001
	Enjoying hobbies	147	52.3	28	20.9	113	76.9	12.582	7.144	22.161	≤ 0.001
	Satisfied with QoL	155	54.0	20	15.2	126	81.3	24.331	13.037	45.409	≤ 0.001
CCS	Abdominal swelling or cramps	79	28.2	76	37.8	64	81.0	7.018	3.736	13.181	≤ 0.001
	Weight loss	50	17.9	102	44.3	37	74.0	3.572	1.803	7.074	≤ 0.001
	Bowel control	160	56.9	35	28.9	106	66.3	4.823	2.892	8.046	≤ 0.001
	Digesting food	125	44.5	46	29.5	99	79.2	9.105	5.242	15.817	≤ 0.001
	Diarrhoea	115	40.9	59	35.5	81	70.4	4.321	2.591	7.204	≤ 0.001
	Appetite	111	38.9	53	30.5	91	82.0	10.388	5.805	18.587	≤ 0.001
	Appearance	202	73.7	9	12.5	128	63.4	12.108	5.692	25.756	≤ 0.001
	Difficulty urinating	49	17.6	108	47.2	31	63.3	1.930	1.021	3.645	0.041
	Urinary frequency	139	49.6	62	44.0	80	57.6	1.728	1.077	2.773	0.023
	Urinary leakage	94	33.8	78	42.4	60	63.8	2.398	1.437	4.003	0.001
Symptom	Breathing difficulties	68	24.5	85	40.5	58	85.3	8.529	4.129	17.619	≤ 0.001
	Changes in taste	49	17.8	96	42.3	45	91.8	15.352	5.340	44.132	≤ 0.001
	Concentration	113	42.5	51	33.3	85	75.2	6.071	3.526	10.455	≤ 0.001
	Constipation	102	37.2	74	43.0	66	64.7	2.428	1.464	4.027	0.001
	Dry congested nose	81	29.7	80	41.7	60	74.1	4.000	2.253	7.100	≤ 0.001
	Dry/itchy/sore skin	110	40.4	66	40.7	75	68.2	3.117	1.873	5.186	≤ 0.001
	Feeling swollen	54	20.1	91	42.3	45	83.3	6.813	3.170	14.643	≤ 0.001
	Hot flushes	59	21.1	101	45.9	43	72.9	3.166	1.683	5.959	≤ 0.001
	Irritability	109	39.4	60	35.7	82	75.2	5.467	3.194	9.356	≤ 0.001
	Memory loss	126	45.7	56	37.3	86	68.3	3.609	2.189	5.951	≤ 0.001
	Mood swings	111	40.2	58	35.2	85	76.6	6.031	3.503	10.383	≤ 0.001
	Sore/dry mouth	62	22.2	93	42.9	51	82.3	6.182	3.055	12.509	≤ 0.001
	Tingling in hands/feet	131	47.0	63	42.6	80	61.1	2.116	1.311	3.417	0.002
SDI	Independence	47	16.7	106	45.3	41	87.2	8.252	3.373	20.185	≤ 0.001
	Domestic chores	92	32.6	76	40.0	72	78.3	5.400	3.041	9.589	≤ 0.001
	Personal care	27	9.6	121	47.8	24	88.9	8.727	2.563	29.718	≤ 0.001
	Care of dependents	28	10.5	111	46.4	26	92.9	14.991	3.480	64.584	≤ 0.001
	Family difficulties with support	18	6.7	124	49.4	15	83.3	5.121	1.447	18.127	0.005
	Benefits	28	10.4	115	47.5	23	82.1	5.080	1.870	13.802	0.001
	Financial difficulties	71	25.4	89	42.8	57	80.3	5.444	2.854	10.386	≤ 0.001
	Financial services	47	16.9	107	46.3	37	78.7	4.288	2.036	9.031	≤ 0.001
	Work difficulties	27	10.4	112	48.3	24	88.9	8.571	2.512	29.253	≤ 0.001
	Future plans	49	17.9	103	45.8	40	81.6	5.264	2.439	11.360	≤ 0.001
	Family communication	52	18.0	107	45.1	44	84.6	6.682	3.016	14.806	≤ 0.001
	Communication with others	38	13.1	116	46.0	35	92.1	13.678	4.100	45.633	≤ 0.001
	Sexual concerns	74	28.7	86	46.7	48	64.9	2.104	1.204	3.677	0.008
	Family planning	10	4.4	114	52.1	8	80.0	3.684	0.765	17.744	0.083
	Body image	80	28.0	85	41.3	63	78.8	5.275	2.886	9.644	≤ 0.001
	Isolation	72	25.3	89	41.8	60	83.3	6.966	3.540	13.709	≤ 0.001
	Mobility/transport	42	14.6	111	45.1	40	95.2	24.324	5.751	102.888	≤ 0.001
	Living arrangements	36	12.4	121	47.6	30	83.3	5.496	2.211	13.660	≤ 0.001
	Recreational activities	83	28.6	79	38.2	73	88.0	11.828	5.769	24.249	≤ 0.001
	Travel plans	89	31.2	72	36.7	75	84.3	9.226	4.864	17.502	≤ 0.001

### Prevalence of survivorship issues

Table 2 presents the frequency of physical, psychological and social issues reported by participants on the EuroQOL 5D-5L, FACT-C, SDI and stand-alone items. The most prevalent survivorship issues reported by survey respondents were dissatisfaction with body image (73.7%, *n* = 202), fatigue (68.4%, *n* = 197), dissatisfaction with sexual function (65.8%, *n* = 102), sleep disturbance (59.0%, *n* = 167) and difficulty finding fulfilment in work (57.1%, *n* = 152). The survivorship issues associated with the highest likelihood of reporting poorer QoL were feeling ill (OR 39.9; 12.3%, *n* = 32), family members’ difficulty accepting their loved ones’ diagnosis (OR 26.3; 20%, *n* = 56), dissatisfaction with QoL (OR 24.3; 54%, *n* = 155), difficulties with transport (OR 24.3; 14.6%, *n* = 42), nausea (OR 21.1, 13.3%, *n* = 33), difficulty enjoying life (OR 19.2; 47.2%, *n* = 133) and difficulties with self-care activities (OR 16.8, 6.1%, *n* = 18). The only issue which was not associated with an increased likelihood of reporting poorer QoL was family planning (Chi Square = 3.00; *p* = 0.083).

### Impact of survivorship issues

During interviews, few participants discussed ongoing challenges with prevalent survivorship issues identified in the initial questionnaire, such as fatigue, sleep disturbance, and sexual function. Interview narratives related to the impact of survivorship issues focused predominantly on less prevalent issues of bowel dysfunction (28.2–56.9%), peripheral neuropathy (47.0%), fear of recurrence (51.3%), financial (25.4%), employment (10.4–56.5%) and impacts on the family (6.7–34.3%). The impact of CRC survivorship issues is illustrated in three themes:

1. *The Vestiges of Colorectal Cancer: Loss and Control,*
2. *The Shadow of Colorectal Cancer: Fear and Vigilance,*
3. *Living Beyond Colorectal Cancer: Impact and Benefit.*

Subthemes within each theme represented a balance between living with the impact of cancer and its treatment (*loss, fear, impact*) and striving to contextualise, reframe and understand the consequences of cancer and its treatment (*control, vigilance, benefit*). Participant quotations (Q), illustrating each theme are presented in Table 3, and are referenced within the results to support interpretation.

**Table 3:** Participant quotations illustrating survivors’ QoL and experience of survivorship issues

Theme 1: The vestiges of colorectal cancer—loss and control	
Subtheme 1.1: Living with loss	
Q1.1	*“I have very finicky work, small bolts and nuts or in awkward places … when it is cold, I have to make doubly sure, be careful, don’t drop this, because it would drop somewhere, it might never again be found.” [RSM048]*
Q1.2	*“I could fall over easily if my foot catches in anything at all. If there’s a stone up, a rock maybe, above the level of the ground and I just catch it, I could go off balance and fall. I’m falling all over the place… every time I go out into the garden unless I’m careful.” [PCM015]*
Q1.3	*“I can’t drive anymore, which I really miss … I feel my whole independence is gone and that’s the saddest thing for me anyway. [My husband] is great … he drives me everywhere, he brings me to all my appointments, and we do the shopping together, things that he has never had to do … but he does everything now you see … anytime I go off for a wedding I’ll be gone before the night is even half-finished, I’d stay for the meal … my legs would be paining me under the table and [my husband], he’d just take me home, because it’s just not worth it. So, all that is kind of stopped … It makes you feel that you’re being left behind.” [ESM073]*
Q1.4	*“I have constant diarrhoea, I would have five, six, maybe even seven bowel movements a day, so I never like to be too far from a toilet … and that has affected my confidence in going out, it would make me more inclined to stay in … I am probably giving up on a social life sooner than I should be.” [RSM028]*
Q1.5	*“I'm always conscious of it because it’s very obvious. No matter what I wear, I feel I can’t hide the stoma, especially if I'm wearing something lighter than this. You can actually see the ridge of the stoma, the opening because it’s actually sitting up on top of the hernia … so ascetically it would bother me a little bit.” [RCM001]*
Q1.6	*“It’s permanent … [I told myself], it’s the bag or the box, so when it’s that choice, of course, you choose life.” [ESM087]*
Q1.7	*“I had no sensation … you don’t realise you're going to the toilet. I had an ileostomy, as they call it, which is very high up and you don’t actually realise half the time that you're going … I could wake-up in the morning, and you’re a baby again, it might have come away.” [ESM036]*
Subtheme 1.2: Striving to regain, maintain and reconceptualise control	
Q1.8	*“I have to manage that [diet] myself, on a trial of hit and miss. There’s been no shortage of effort in [Hospital] to assist me on that. But the knowledge basis is very shallow, I would say … the individualisation of treatment seems to be a recurring theme.” [ESM043]*
Q1.9	*“Before it was reversed to what I have now [ileostomy], that was a strenuous time, because it wasn’t getting any better no matter what was [done] food wise … I was fighting so much with my body to not have to get that done … but I had to understand myself; I wasn’t going to have any quality of life at all, I’d never be able to go out in any sense.” [RCM049]*
Q1.10	*“My specialist subject is all accessible toilets within an hour’s walk of my home. I know all the toilets in town, anywhere I go, even subconsciously at this stage.” [ESM036]*
Q1.11	*“I find I can’t take things in as well as before. It takes me longer if I’ve to read a complicated report now … I’m a bit more forgetful, but sure that’s probably age.” [ESM005]*
Q1.12	*“Well there is, yes, a slight, a loss of energy but a certain amount of that is down to age too, I'm going to be seventy now in two months, so you have to accept that you're not as fit as you were when you were twenty-five.” [ESM006]*
Q1.13	*“When I was undergoing the chemo, I had problems sleeping, so I would spend a lot of time down in the kitchen. I baked a lot of bread at four o’clock in the morning, and four o’clock in the morning is still a time when I’d wake up … maybe half the nights of the week and I’m back in the kitchen shall we say. I’m not physically in the kitchen, but mentally I can feel that chill, those cold thoughts.” [ESM043]*
Theme 2: The shadow of colorectal cancer—fear and vigilance	
Subtheme 2.1: Living in the shadow of colorectal cancer	
Q2.1	*“I’d probably end up in a wheelchair if I got more chemo. So that’s my biggest worry that it would come back, and I couldn’t have treatment … every time I feel ‘God will I be called back? … My brother that died, his death had an awful effect on me because he had a terrible death.” [ESM073]*
Q2.2	*“While I had no symptoms of bowel cancer before the surgery, I have had all the symptoms that you are told to look out for since the surgery. That is, I have constant diarrhoea, I would have maybe five, six, maybe even seven bowel movements a day.” [RSM028]*
Q2.3	*“You'll never have a headache again, it’ll be a brain tumour … you'll think worst case scenario, and that is me, I have turned into that person … you wake in the morning, how am I? … Am I okay? … Is that different from one I've had before? … Which of the symptoms is that now? Which thing is that now?” [ESM036]*
Subtheme 2.2: Striving for vigilance	
Q2.4	*“Every time it comes up that you’ve to go back for a scan or a colonoscopy, or whatever, you worry a little bit that they're going to find something again. You see somebody who’s never had cancer doesn’t have that worry, but once you’ve had it you have that worry all the time that they're going to find something else, that it's going to appear again or its spread somewhere else.” [ESM006]*
Q2.5	*“I’m certainly more keen on maintaining a position where perhaps the symptom might come up and you notice very early … and not doubting myself … I’m making a note of something and make sure that I am seeing somebody.” [ESM043]*
Q2.6	*“You can get a bit paranoid and then you start thinking you’re bothering people. Ringing up the nurses to get my bloods done … just to check, all that kind of thing.” [ESM043]*
Q2.7	*“I've spent a fortune on consultants, my doctor is fed up listening to me and it just turns you into someone like that.” [ESM036]*
Theme 3: Living beyond colorectal cancer—impact and benefit	
Subtheme 3.1: Living with the impact of colorectal cancer	
Q3.1	*“[Social welfare] is €188 whether you like it or not and then you’ve to do the juggling … I need €25 of electric, I need €25 worth of gas, I need €10 on the bin to be collected, I have to pay €50 a week rent. And that is reality … it's like your diet, people saying eat organic meat … change my diet … I can't afford it.” [RSM027]*
Q3.2	*“I suppose… one of my fears when I was diagnosed with cancer was ‘Oh my god, will I get back to work or…?’ It’s all ‘Will I? Will I? Will I?’ So, I was delighted to get back to work, absolutely thrilled to get back to work.” [ESM087]*
Q3.3	*“I went back to work maybe two days a week, I finished my treatment in March, and I didn’t go back to work until August … [My] local doctor advised me strongly to take as much time off as I could because once you were back at work, you were back at work.” [ESM005]*
Q3.4	*“I was coming home, I would have my dinner, I'd go to bed, and I'd drag myself out of the bed in the morning, so I just couldn’t hack it.” [ESM036]*
Q3.5	*“I miss my long-haul holidays … there's the money side of the social life as well and the holidays … since I did the course and starting [teaching], I've a new group of friends … so that’s a whole new thing that’s going to open. Now I joined the library, I never joined a library before in my life.” [ESM036]*
Q3.6	*“I only heard afterwards that [my husband] was very worried. And my children, they think I’m invincible and the idea of it, they were so shocked.” [ESM121]*
Q3.7	*“I tell you cancer is a very lonely place because no matter how many people are around you and want to help, you’re in it on your own … When you’re sick yourself you know how you’re getting along, you know how you’re feeling, you know that you just need to rest … You don’t know how the other person is; you don’t know if they’re feeling stronger today or they’re feeling weaker today or what you can do to help them. Now my brother is like me, ‘Go away, don’t bother me,’ when I’m sick I really want to be on my own … but I found I worried much more about him.” [PCM026]*
Q3.8	*“[I] don’t have a partner anymore, [I’m] single and not really into the whole dating thing, I don’t know if that’s got anything to do with cancer and the stoma, I don’t know, maybe?” [ESM087]*
Q3.9	*“Fortunately, I’m in a long-term relationship. Again, if I was younger, and perhaps was dating, I’m not quite sure how I’d handle that.” [ESM043]*
Q3.10	*“When people look at you, they think ‘Oh god look at your feet, your feet look perfect,’ and you’d say [it’s peripheral neuropathy], and they can’t understand, they think it’s all in your head.” [ESM073]*
Q3.11	*“If you hurt or break your arm … you can freely talk about it, but to start talking to people, even people that are very close to you, about your bowel movement or whatever, nobody wants to know because the toilet is a place that’s just for one at a time.” [RCM049]*
Subtheme 3.2: Striving to find benefits in the experience of cancer	
Q3.12	*“I don’t know how it affects other people, there was a woman down the road there, she’s dead now, God rest her, but she got neuropathy after cancer, she could hardly walk, she got it really bad. I’m counting my blessings; it’s not stopping me getting around.” [PCM015]*
Q3.13	*“I do some unofficial home visits to neighbours and friends who are diagnosed with colorectal cancer and use a stoma. I tell them what works for me and keep the chat very upbeat and positive. I tell them they can ask me anything and they do because we can be empathetic.” [RCM001]*
Q3.14	*“I did put the word out in the oncology ward, I told them in there … that if anybody wanted to talk about it or was due to go through it and wanted to talk about it on a one-to-one basis that I would be quite willing to do that.” [RCM013]*
Q3.15	*“I have told all of my friends, a lot of whom were invited [to BowelScreen] but hadn’t bothered filling in the form … so I've been advocating to every one of the age to make sure that they do that.” [PCM018]*
Q3.16	*“I think that if I had had somebody coming around to do the changing of the bag for me all the time I'd have developed a poor me attitude. I reckon that it was better for me that I was left to do it myself.” [RCM013]*
Q3.17	*“Just waiting for your life to start [and] get back to where you were. Sometimes you think you’re never going to get there, there is nothing that’s going to take this away from you … I have to have hope and then [my husband] gives me great hope, he keeps saying ‘there will, there’ll be something that will come on the market that will help.’” [ESM073]*

### Theme 1: the vestiges of colorectal cancer—loss and control

#### 1.1: Living with loss

*Living with loss* referred to the loss of control associated with bowel dysfunction and loss of sensation associated with peripheral neuropathy.

Peripheral neuropathy was described as a frustrating, difficult to manage symptom, with rapid fluctuations between freezing and burning sensations, numbness, and pins and needles in the hands and feet. While participants anticipated peripheral neuropathy during treatment, many who discussed ongoing limitations in survivorship, described their lack of preparation for its potentially chronic, irreversible nature. One survivor described how pain associated with peripheral neuropathy contributed to a loss of independence and withdrawal from social and family activities [Q1.1–Q1.3].

Narratives of bowel dysfunction highlighted survivors’ embarrassment arising from faecal urgency, frequency, and incontinence, which hindered social activities and contributed to social isolation, depression and sleep disturbance [Q1.4]. The prospect of a temporary or permanent ostomy was feared by survivors, due to aesthetic, social and self-management concerns [Q1.5]. For some, it was a price to pay for survival [Q1.6]. Although most ostomates became proficient in its management, many relayed initial struggles, including equipment failures, accidents and loss of bowel control [Q1.7].

#### 1.2: Striving to regain, maintain and reconceptualise control

*Striving to Regain, Maintain and Reconceptualise Control* described the self-management strategies devised by CRC survivors to manage physical and psychological survivorship issues. While managing bowel dysfunction was a key focus of discussion within this subtheme, participants also described approaches used to reframe or reconceptualise the impact of survivorship issues.

Regaining control was a process of trial and error with variable success, some questioned if they would ever be ‘*normal*’ again, describing significant continuing anxiety and social isolation associated with bowel dysfunction. One survivor requested that his ileostomy be reinstated as his bowel dysfunction remained debilitating several years after its original reversal [Q1.8–1.9]. Some accepted bowel dysfunction as a new normal, but described the extensive planning needed to self-manage bowel dysfunction to allow them to maintain their social life, including toilet mapping, planning ahead and menu shopping [Q1.10]. Several survivors attributed a selection of symptoms to ageing, including fatigue, sleep disturbance and sexual dysfunction [Q1.11–Q1.12]. Younger survivors associated sleep disturbances and exacerbations of fatigue with anxiety or stress [Q1.13].

### Theme 2: the shadow of colorectal cancer—fear and vigilance

#### 2.1: Living in the shadow of colorectal cancer

*The Shadow of Colorectal Cancer* comprised the psychological consequences of diagnosis and treatment. The possibility of recurrence was the most dominant fear in the aftermath of treatment. Fear of recurrence was about fear of mortality, and a fear of re-living physical and psychological impact of diagnosis and treatment and effects of treatment, including peripheral neuropathy and bowel dysfunction [Q2.1].

Fear of recurrence resulted in a heightened state of alert among survivors. For many, the physical symptoms experienced during and after treatment were reminiscent of those preceding diagnosis. As a result, negligible changes in symptoms were often magnified. Several factors sustained fear of recurrence, including shorter time since treatment, presence of genetic risk factors and awareness of friends or family members’ diagnosis or death from cancer [Q2.2–Q2.3].

#### 2.2: Striving for vigilance

To mediate fear of recurrence, survivors remained vigilant to the risk of recurrence through routine surveillance and self-awareness. Survivors described the dualities of vigilance, in how it could support coping, but also trigger anxiety. Surveillance was reassuring, enhancing the potential for early detection of recurrence. However, attending hospital appointments for surveillance triggered distressing memories of cancer. While self-awareness was empowering, some feared it could be misinterpreted as paranoia and could disenfranchise survivors were they perceived to ‘*cry wolf*’. Self-awareness also had financial implications, as survivors sought investigation of symptoms via private healthcare to avoid potential delays in the public health system [Q2.4–Q2.7].

### Theme 3: living beyond colorectal cancer—impact and benefit

#### 3.1: Living with the impact of colorectal cancer

*Living with the impact of colorectal cancer* encompassed the social domain of survivorship, including its impact on employment, financial well-being and relationships.

The financial impact of cancer persisted into the survivorship period for many participants; several who remained on long-term leave from employment spoke of the inadequacy of illness-related social welfare payments to meet the financial costs of living after cancer, including costs of transport and parking associated with hospital appointments and access to nutritionally balanced meals [Q3.1].

For a small cohort, returning to employment was a milestone in recovery, achieved with the support of their general practitioner and employer. Those who successfully returned to work emphasized the need to return on a phased basis [Q3.2–Q3.3]. Some described returning to full-time work quickly, motivated by the opportunity to regain a semblance of normality. However, many of this group described difficulties managing the physical consequences of treatment, including fatigue, pain and peripheral neuropathy and withdrew from the workforce voluntarily or involuntarily [Q3.4]. Invariably, the financial impact of cancer lead to a tapering of hobbies and routine activities. Despite regret at these losses, one survivor described the discovery of new opportunities, interests and social outlets [Q3.5].

Participants described the impact of cancer on their families, including anxiety and distress, and their transitions to new roles as advocates, caregivers and homemakers, supporting the survivor. Family well-being was a source of concern for survivors; several feared becoming a burden on their family if they became ill again or required informal care in the home [Q3.6–Q3.7]. While the experience of cancer appeared to strengthen family relationships for the most part, a small number of participants attributed breakdowns in their relationships to cancer. Younger survivors revealed particular concerns about establishing new relationships in the future [Q3.8–Q3.9].

While friends, neighbours and work colleagues were often an extension of the family unit, providing essential support during treatment, many described a lack of understanding and awareness of the long-term implications of cancer among their social circles. Participants compared the invisibility of their CRC, to other cancers, associated with alopecia and significant changes in appearance. The invisibility and stigma of physical survivorship issues such as bowel dysfunction and peripheral neuropathy created a barrier to support from social networks and was a source of distress for some [Q3.10–Q3.11].

#### 3.2: Striving to find benefits in the experience of cancer

Despite the overwhelming challenges of CRC survivorship, many survivors seemed able to derive benefit from the experience. Participants who described severe difficulties with bowel dysfunction and peripheral neuropathy spoke of reframing their circumstances and feeling gratitude for their lives, often comparing themselves to others who had a more difficult diagnosis or experience [Q3.12]. An extension of gratitude for life was a desire to help others and give back to the services that helped them, and to support people living with cancer, through peer-support, participation, and engagement in research, and promoting awareness [Q3.13–Q3.15].

Many survivors confronted the physical, psychological and social impact of their disease, and described using either self-reliant or support-reliant coping strategies; no participant described using self-reliant and support-reliant coping strategies in tandem. Survivors who used supportant strategies placed importance upon the support of family, friends, healthcare professionals and cancer advocacy organisations. Those who used self-reliant strategies expressed the desire to deal with the experiences in their own way. Although the importance of healthcare professionals was acknowledged by members of this group, these survivors were more pragmatic in learning to live with and beyond their disease [Q3.16–Q3.17].

## Discussion

This mixed methods study has provided insight into the prevalence, nature and impact of physical, psychological, and social survivorship issues experienced by CRC survivors up to five years following diagnosis. This study adds to an emerging body of mixed methods literature exploring the QoL outcomes of people living with and after cancer, providing a nexus between the qualitative and quantitative literatures of cancer survivorship. Integration of the quantitative and qualitative findings highlights a dichotomy between the prevalence and impact of survivorship issues on CRC survivors’ day-to-day lives. While participants’ QoL outcomes were comparable to population norms, more than half of the sample reported dissatisfaction with their QoL, experiencing a range of survivorship issues. Survivors lived with the impact of cancer and its treatment (loss, fear, impact), and strived to contextualise, reframe and understand the consequences of cancer and its treatment (control, vigilance, benefit).

While negative body image, fatigue, sexual dysfunction, sleep disturbance and impact on work were the most common survivorship issues reported by participants, the converging findings of crosstabulations and interview analysis suggests these were not necessarily the issues which had the greatest impact on cancer survivors’ QoL. In keeping with Lim, Laidsaar-Powell [45], interview data suggested that physical issues of bowel dysfunction and peripheral neuropathy impacted survivors’ social, psychological and functional QoL. In this study, the broad topics of interview discussions did not directly reflect the quantitative issues associated with a greater likelihood of reporting poorer QoL, including feeling unwell, having difficulties enjoying life and ability to self-care. While these issues were less prevalent in the quantitative study, interviewees directly attributed feeling unwell, having a compromised social life and losing independence to bowel dysfunction and peripheral neuropathy. The convergence between these findings suggests that less prevalent functional issues have the potential to compromise cancer survivors’ day-to-day lives across multiple domains. During interviews, functional issues appeared to be attributed to physical survivorship issues and were most likely to be associated with poorer QoL among CRC survivors.

More common physical and functional effects, such as fatigue and sexual dysfunction were less commonly discussed by participants during interviews, and more often attributed to ageing processes, comorbidity, or stress, and were not associated with psychological or social distress. While the attribution of symptoms to ageing diverges from Lim, Laidsaar-Powell [45], it aligns with previous qualitative studies exploring CRC survivorship [11, 26]. People of all ages living with and after a cancer diagnosis experience a range of chronic physical, psychological, and social issues as a result of cancer and cancer treatments [46, 47]. While older adult cancer survivors are likely to maintain independence in instrumental activities of daily living, they may be more likely to experience poorer health outcomes and chronic conditions [48]. Increasing multimorbidity and issues of social support enhance the complexity of healthcare provision for this population, meaning older adults may be disenfranchised within specialist care services [49, 50]. Misconceptions regarding the origin of survivorship issues may contribute to under-reporting of these issues, creating an additional barrier to supportive care and self-management of survivorship issues which are amenable to intervention. As understanding of the specific needs and experiences of older adults with cancer develops, it is important to understand how perceptions of ageing in the context of survivorship can influence the experience, reporting and management of survivorship issues.

The comparability of CRC survivors’ QoL with US and Austrian population norms are potentially attributable to cultural differences related to health and healthcare. Nevertheless, these findings are consistent with previous prospective studies [3, 4, 48]. The sequential mixed methods approach adopted in this study provides further information to support interpretation of these findings. As in previous studies, participants of the current study described various strategies to reframe and cope with physical, psychological and social impacts of CRC, including attribution of survivorship issues to ageing; benchmarking their wellbeing against others with cancer; engaging with self-monitoring and surveillance; and deriving benefit from the experience of cancer [11, 22]. Despite these strategies, psychosocial survivorship issues, including worry about cancer, anxiety and depression were prevalent, affecting half and one-third of participants, respectively. While surveillance and self-awareness were notable strategies for coping with fear of recurrence, they also possessed the potential to contribute to anxiety, reflecting the findings of previous studies [11, 22, 51].

**Fig. 1:**
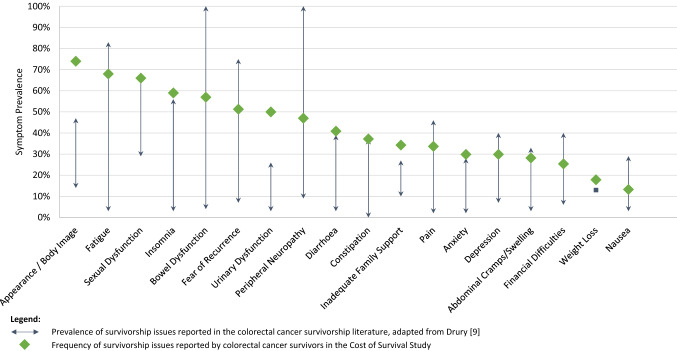
Comparison of prevalence of survivorship issues among colorectal cancer survivors in the current study and those reported in previous studies, adapted from Drury [9]

This study adds to a body of literature which reports wide variation in physical, psychological and social survivorship issues in CRC survivorship (Fig. 1). Variance in survivorship issues may be attributed to varying structure of questions and timeframes examined by QoL instruments. For example, timeframes examined by instruments used in this study ranged between one day (EuroQOL 5D-5L), one week (FACT-C) and one month (SDI). Furthermore, variance between similar issues on the FACT-C and SDI identified in this study (e.g. *I like the Appearance of my Body—*FACT-C, 74%; and *Have You Had Any Difficulty Concerning Your Appearance or Body Image?*—SDI, 28%) may be attributed to both the timeframe examined, the structure of the statement/question and response scale and varying terminologies used. Disparities in QoL outcomes measured on standardised and single-item global instruments may be related to cultural factors which mediate negative health-related outcomes [38, 52]. While single-item measures may provide valid estimates of life satisfaction, and are more sensitive to state variance compared to multi-item scales, they are less likely to capture trait variance, and may partly confound measurement issues [53]. While the QoL instruments selected for the current study represent some of the most valid, reliable and widely used QoL instruments, they are orientated toward the assessment of difficulties during cancer treatment, and may overlook the specific challenges of survivorship [54]. Nevertheless, the combination of quantitative and qualitative research methods in the current study enhances the meaning and understanding of complex QoL concerns in cancer survivorship, identifying additional QoL concerns and coping strategies used by survivors to reframe their experiences of illness and expectations of QoL.

## Limitations

The results of this study must be interpreted with consideration of several limitations. Firstly, since the article reports two-sample comparison with previously reported population norms, our sample is not matched in terms of age and gender. Secondly, the dichotomisation of the FACT-C score for chi-square analysis based on the sample median limits the generalisability of the data. The cross-sectional nature of the quantitative and qualitative phases of this study, and the ethnic homogeneity of the sample limits the generalisability of the findings.

## Conclusions

The findings of this study demonstrate that although cancer survivors report positive QoL outcomes, many may experience distressing physical, psychological and social effects. In preparing CRC survivors for the potentially chronic issues associated with cancer and its treatment, there may be a preponderance among healthcare professionals to focus on highly prevalent issues. This study highlights that the most prevalent survivorship issues are not always the most impactful, and factors associated with the highest likelihood of poorer QoL outcomes may not be directly identified as survivorship issues, but may be associated with distressing survivorship issues, such as bowel dysfunction and peripheral neuropathy which affect survivors’ QoL in multiple domains. Supporting survivors to live with and overcome the challenges of survivorship issues is complex. Supportive care and self-management interventions must be tailored to address survivors’ individual needs and preferences for support; holistic person-centred assessment offers opportunities to identify and understand survivorship issues which have the greatest impact on CRC survivors’ wellbeing. Interventions which target multi-focal survivorship issues through supportive care and supported self-management which facilitate survivors to develop coping and problem-solving skills may be a strategy to address and alleviate the multi-dimensional impact of survivorship issues.

## Supplementary Information

Below is the link to the electronic supplementary material.

Supplementary file1 (DOCX 40 KB)

## Data Availability

The datasets generated during and/or analysed during the current study are not publicly available due to data protection and privacy concerns. Excerpts of data are available from the corresponding author upon reasonable request.
