# Supplementary material for: Prevalence vs impact: a mixed methods study of survivorship issues in colorectal cancer
Source: Qual Life Res. 2021 Aug 21;31(4):1117–34. doi: 10.1007/s11136-021-02975-2 (PMC8960628; doi:10.1007/s11136-021-02975-2)
Supplement: Supplementary file 1 — Supplementary file1 (DOCX 40 KB) [file 11136_2021_2975_MOESM1_ESM.docx]

Prevalence vs Impact: A mixed methods study of survivorship issues in colorectal cancer

*Supplementary Material*

*Quality of Life Research*

Dr. Amanda Drury^1^, Professor Payne Sheila^2^, Professor Brady Anne-Marie^3^

^1^ Assistant Professor, School of Nursing, Midwifery and Health Systems, University College Dublin, Belfield, Dublin 4. [amanda.drury@ucd.ie](mailto:amanda.drury@ucd.ie). Twitter: @druryal.

^2^ Emeritus Professor, International Observatory on End of Life Care, Division of Health Research, Lancaster University, Lancaster, LA1 4AT, UK. [s.a.payne@lancaster.ac.uk](mailto:s.a.payne@lancaster.ac.uk)

^3^ Chair of Nursing and Chronic Illness, School of Nursing & Midwifery, Faculty of Health Sciences, Trinity College Dublin, 24 D'Olier Street, Dublin 2, Ireland, D02 T283. [abrady4@tcd.ie](mailto:abrady4@tcd.ie)

**Corresponding Author:**

Amanda Drury, School of Nursing, Midwifery and Health Systems, University College Dublin, Belfield, Dublin 4. E-mail: [amanda.drury@ucd.ie](mailto:amanda.drury@ucd.ie). Telephone: +353 1 716 7777.

**Funding:**

This research was supported by funding from the Health Research Board of Ireland, grant number HPF.2014.715.

# Supplementary Appendix 1

Supplementary Appendix 1 Prevalence of survivorship issues among colorectal cancer survivors reported in the literature, adapted from Drury [9]

# Supplementary Appendix 2

Supplementary Appendix 2 Results of two-proportion Z-tests to compare the proportion of colorectal cancer survivors reporting difficulties on EuroQol 5D-5L items with general population norms

| EuroQOL Subscale | Current Study | | *Hobbins et al. [20] (Ireland)* | | | | | | | *Kind et al. [21] (UK)* | | | | | | |
| --- | --- | --- | --- | --- | --- | --- | --- | --- | --- | --- | --- | --- | --- | --- | --- | --- |
|  | ***n*** | ***%*** | ***n*** | ***%*** | ***Difference***  ***(%)*** | ***95% CI***  ***Lower (%)*** | ***95% CI***  ***Upper (%)*** | ***Z*** | ***p*** | ***n*** | ***%*** | ***Difference***  ***(%)*** | ***95% CI***  ***Lower (%)*** | ***95% CI***  ***Upper (%)*** | ***Z*** | ***p*** |
| Mobility | 85 | 28.8 | 245 | 21.7 | 7.2 | 2.0 | 12.3 | 3.792 | ≤ 0.001 | 623 | 18.4 | 10.5 | 5.3 | 15.6 | 5.942 | ≤ 0.001 |
| Self-Care | 18 | 6.1 | 71 | 6.3 | -0.2 | -2.9 | 2.5 | -0.182 | 0.572 | 144 | 4.2 | 1.8 | -0.9 | 4.6 | 2.008 | 0.022 |
| Usual Activities | 108 | 36.7 | 217 | 19.2 | 17.5 | 12.0 | 23.1 | 9.352 | ≤ 0.001 | 551 | 16.2 | 20.5 | 15.0 | 26.0 | 11.970 | ≤ 0.001 |
| Pain/Discomfort | 98 | 33.7 | 458 | 40.5 | -6.8 | -12.2 | -1.4 | -3.124 | 0.999 | 1117 | 32.9 | 0.8 | -4.7 | 6.2 | 0.369 | 0.356 |
| Anxiety/Depression | 88 | 29.9 | 249 | 22.0 | 7.9 | 2.7 | 13.2 | 4.166 | ≤ 0.001 | 710 | 20.9 | 9.0 | 3.8 | 14.3 | 4.898 | ≤ 0.001 |

# Supplementary Appendix 3

Supplementary Appendix 3 Results of two-sample t-tests to compare EuroQOL VAS and FACT Subscale scores in the current sample with general population norms

|  | Current Study | | | | *Hobbins et al. [20] (Ireland)* | | | | | | | | | *Kind et al. [21] (UK)* | | | | | | | | |
| --- | --- | --- | --- | --- | --- | --- | --- | --- | --- | --- | --- | --- | --- | --- | --- | --- | --- | --- | --- | --- | --- | --- |
|  | ***n*** | ***Mean*** | ***SD*** | ***SE*** | ***n*** | ***Mean*** | ***SD*** | ***SE*** | ***Difference*** | ***95% CI Lower*** | ***95% CI Upper*** | ***t*** | ***p*** | ***n*** | ***Mean*** | ***SD*** | ***SE*** | ***Difference*** | ***95% CI Lower*** | ***95% CI Upper*** | ***t*** | ***p*** |
| EuroQOL VAS | 287 | 81.2 | 16.1 | 1.0 | 1131 | 79.9 | 14.9 | 0.4 | 1.3 | -0.7 | 3.3 | 1.298 | 0.194 | 3395 | 82.5 | 17.0 | 0.3 | -1.3 | -3.3 | 0.7 | -1.249 | 0.212 |
|  | ***Current Study*** | | |  | ***Brucker et al. [19] (USA)*** | | | | | | | | | ***Holzner et al. [3] (Austria)*** | | | | | | | | |
| FACT-G | 254 | 89.3 | 15.5 | 1.0 | 1075 | 80.1 | 18.1 | 0.6 | 9.2 | 6.8 | 11.6 | 7.478 | ≤ 0.001 | 926 | 86.5 | 15.2 | 0.5 | 2.8 | 0.7 | 4.9 | 2.590 | 0.010 |
| PWB | 264 | 24.6 | 4.3 | 0.3 | 1075 | 22.7 | 5.4 | 0.2 | 1.9 | 1.2 | 2.6 | 5.317 | ≤ 0.001 | 926 | 24.9 | 4.1 | 0.1 | -0.9 | -0.3 | -1.3 | -1.037 | 0.300 |
| SWB | 278 | 23.3 | 5.4 | 0.3 | 1075 | 19.1 | 6.8 | 0.2 | 4.2 | 3.3 | 5.1 | 9.548 | ≤ 0.001 | 926 | 20.2 | 5.8 | 0.2 | 3.1 | 2.3 | 3.9 | 7.938 | ≤ 0.001 |
| EWB | 276 | 20.1 | 3.9 | 0.2 | 1075 | 19.9 | 4.8 | 0.1 | 0.2 | -0.4 | 0.8 | 0.640 | 0.552 | 926 | 19.5 | 4.5 | 0.2 | 0.6 | 0.0 | 1.2 | 2.002 | 0.045 |
| FWB | 283 | 21.2 | 6.6 | 0.4 | 1075 | 18.5 | 6.8 | 0.2 | 2.7 | 1.8 | 3.6 | 5.979 | ≤ 0.001 | 926 | 21.4 | 5.5 | 0.2 | -0.2 | -1.0 | 0.6 | -0.510 | 0.610 |
| CCS | 283 | 22.1 | 5.0 |  |  |  |  |  |  |  |  |  |  |  |  |  |  |  |  |  |  |  |
